# COVID-19 Data Analytics Using Extended Convolutional Technique

**DOI:** 10.1155/2022/4578838

**Published:** 2022-11-07

**Authors:** Anand Kumar Gupta, Asadi Srinivasulu, Olutayo Oyeyemi Oyerinde, Giovanni Pau, C. V. Ravikumar

**Affiliations:** ^1^Data Science Research Laboratory, BlueCrest University College, Monrovia, Liberia; ^2^School of Electrical and Information Engineering, University of the Witwatersrand, Johannesburg 2050, South Africa; ^3^Kore University of Enna, Faculty of Engineering and Architecture, Enna 94100, Italy; ^4^Sense, Vellore Institute of Technology, Vellore, Tamil Nadu, India

## Abstract

The healthcare system, lifestyle, industrial growth, economy, and livelihood of human beings worldwide were affected due to the triggered global pandemic by the COVID-19 virus that originated and was first reported in Wuhan city, Republic Country of China. COVID cases are difficult to predict and detect in their early stages, and their spread and mortality are uncontrollable. The reverse transcription polymerase chain reaction (RT-PCR) is still the first and foremost diagnostical methodology accepted worldwide; hence, it creates a scope of new diagnostic tools and techniques of detection approach which can produce effective and faster results compared with its predecessor. Innovational through current studies that complement the existence of the novel coronavirus (COVID-19) to findings in the thorax (chest) X-ray imaging, the projected research's method makes use of present deep learning (DL) models with the integration of various frameworks such as GoogleNet, U-Net, and ResNet50 to novel method those X-ray images and categorize patients as the corona positive (COVID + ve) or the corona negative (COVID -ve). The anticipated technique entails the pretreatment phase through dissection of the lung, getting rid of the environment which does now no longer provide applicable facts and can provide influenced consequences; then after this, the preliminary degree comes up with the category version educated below the switch mastering system; and in conclusion, consequences are evaluated and interpreted through warmth maps visualization. The proposed research method completed a detection accuracy of COVID-19 at around 99%.

## 1. Introduction

The ailment referred to as “the extreme acute breathing syndrome coronavirus 2 (SARS-CoV-2)” was determined by the end of December 2019. Accordant to reports, this disease was originated in China and has become the reason for the disorder referred to as “Corona Virus Disease 2019,” in short, “COVID-19.” The specialized health institution of the USA, the “World Health Organization” (WHO), stated that this disorder is a “deadly disease” in mid-March 2020 [1–5]. As per reviews delivered to date with the aid of using worldwide health organizations, authorities/entities, and governments, the pandemic has affected tens of thousands and thousands of human beings globally. The maximum severe contamination due to COVID-19 is associated with the lungs, which includes pneumonia. The signs and indications of the disorder may range and consist of excessive body temperature (high fever), dyspnea, coryza, and cough. These instances can be normally recognized through the usage of lung X-ray evaluation of the irregularities [6]. This research is the latest in its own field, targets the global pandemic caused by the coronavirus, and helps scholars to implement combat tools and techniques following the proposed method in this research work.

Throughout this hasty period, several scholars have tried to expand numerous transmission gear and diagnosing systems, such as the real-time reverse transcription polymerase chain reaction (RT-PCR), which is still claimed as a vital testing technique to discover extremely severe breathing disease SARS-COV2 [1–3] and in addition to COVID-19. Though RT-PCR is considered to be the best method of screening so far, it still has limitations. The working system of the RT-PCR is complex and cumbersome [2–5]. The input dataset, which has 39,256 X-ray images, was collected from various publicly available dataset repositories such as Kaggle, UCI, Google, and GitHub. This research is purely conducted on the sample datasets collected from these various repositories, which require no consent from human being. Thus, trials were attempted for detecting COVID-19 disease through lung X-ray images which include CT (computed tomography) or lung x-beam photographs. It is said the investigative significance and accurateness of CT lung photographs over RT-PCR in COVID-19 [2] are highly accepted. The discoveries display that a lung CT scans are an excessive sensitivity for the analysis of COVID [7].

## 2. Literature Review

The global impact of the global pandemic caused by the coronavirus is immense. It is thought that Wuhan, China, is where the coronavirus was initially identified [1]. The coronavirus quickly spread around the world. Health officials have been working to limit the virus as cases of the illness it causes have grown sharply. On December 1, 2019, in Wuhan, China, a patient was diagnosed with viral pneumonia. This patient is mentioned in a medical study published in The Lancet Journal and is thought to be the first case of COVID-19 to be recorded. Since then, a worldwide chain reaction of reported pneumonia cases has begun to spread, first in Wuhan and then globally [7–10]. Fever, difficulty breathing, sneezing, and coughing were the main symptoms that were seen in the patients. The World Health Organization (WHO) was notified by the Chinese government of an outbreak of pneumonia cases in Wuhan towards the end of December; 44 instances were reported to the WHO between December 31 and January 3, 2020. The international airport in Wuhan connects the city with foreign nations as well as with other Chinese cities. People with COVID-19 who travel from Wuhan to other cities in China and other countries carry the virus with them. When more people came into contact with the afflicted individuals, the virus was spread [11].

The call for quicker analysis of COVID-19, more than one research carried out to focus on layout answers and clinical records concerning this exceedingly transmittable disease. Some picture identification, examination, clarification, and conclusion strategies were indexed in this segment. The DL (deep learning) method [12] has been projected and has efficaciously received satisfying outcomes in phrases of accuracy in diverse arenas [6, 12]. The instance research of COVID-19 examination of CT scans had been offered with the aid of using authors together with Li et al. [13], Xu et al. [14], Gozes et al. [15], and Shi et al. [16]. Authors [14] mentioned as follows as the COVID-19 eminent shows its traits which can change starting with different varieties of virus-related pneumonitis, such as biological influenza-A pneumonia [5,17,18]. The goal of this research study has become to broaden a preliminary testing outline for COVID-19 with the aid of using automatic respiratory CT scans (CT photographs) of COVID-19, pneumonia, and ordinary instances. They considered 628 CT scans and X-ray test pattern images earlier than expansion, and their version acquired an accuracy of 90%. The writers' approach consists of image preprocessing, dissection of more than one region (patches) accepting V-Net (volumetric network) [19] based on separation version V-Net-IR-RPN [20], that has skilled for pulmonic tuberculosis resolution.

**Figure 1:**
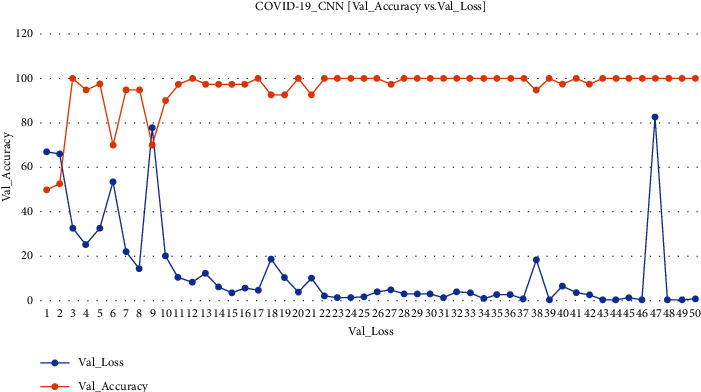
COVID-19 ECNN graph comparing Val_Accuracy vs. Val_Loss.

**Figure 2:**
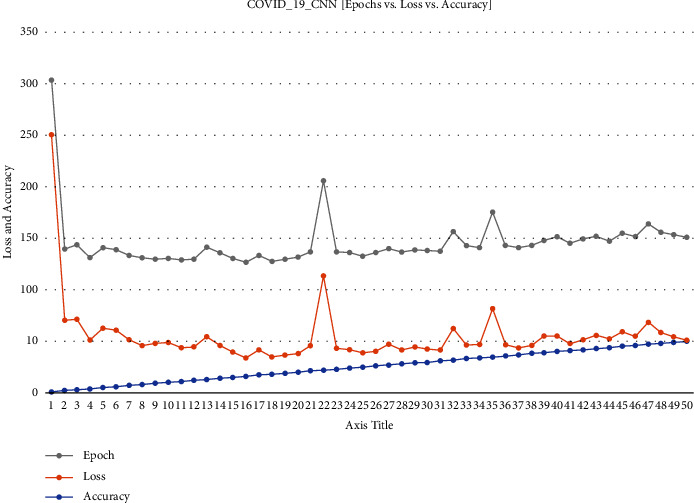
COVID-19 ECNN graph comparing epoch vs. loss vs. accuracy.

Our method includes three (3) essential experiments to assess the overall performance of the predication and determine an effect on the distinctive levels of the procedure. The respective test follows the workflow. The distinction among trials is the dataset used from various repositories [21, 22]. In all occurrences, identical photographs of COVID-19 effective instances were used. Meanwhile, 3 distinctive datasets for poor instances were utilized. In that direction, experiments 1 and 2 included comparing effective vs. poor instances datasets Figure1, and experiment three entails pre-COVID generation images (from 2015–2017), further Table1 explains the comparative analysis of the proposed model (ECNN) with the existing system (CNN) in details through various parameters Figure 2.

Muhammad Irfan et al. developed a combined model by using CT and X-ray imaging concept named as HDNNs (hybrid deep neural networks) to predict severity levels of COVID-19 infectious by classifying the data into 3 classes such as pneumonia, normal, and COVID-19 positive [20]. Yassir Edrees Almalki et al. proposed a unique framework named as COVID Inception-ResNet (CoVIRNet) model, which uses chest X-ray images to assess the infection of COVID-19 naturally. The projected calculation has different initiation remaining blocks that take special care of data by utilizing various profundities highlight maps at various scales, with the different layers [23].

## 3. Methodology

### 3.1. Existing System

The referred existing system has approachable superficial learning strategies, for example, The Convolution Neural Organization and Intermittent Neural Organization. CNN computation drawbacks are as follows:

- Less efficient
- High time complexity
- High execution time
- Highly error prone
- Supports only a small dataset

### 3.2. Proposed System

The proposed research work was carried out by using the available deep learning method convolutional NN (neural network) by using combined methods of AI, ML, and DL and hence was named as extended convolutional neural network (ECNN).

ECNN algorithm advantages are as follows:

- Highly accurate
- Less time consuming
- Less Performance time
- Less error rate
- Big Data Scope

## 4. Experimental Results

The essential thought is to execute, guarantee, and ensure that the coronavirus illness severer impacted job gathered measurements worked in the manner that can force readiness and development from their most memorable viewpoint.

**Figure 3:**
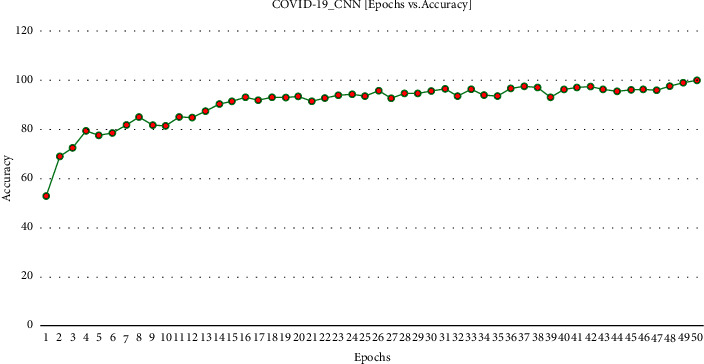
COVID-19 ECNN graph comparing accuracy vs. epoch.

**Figure 4:**
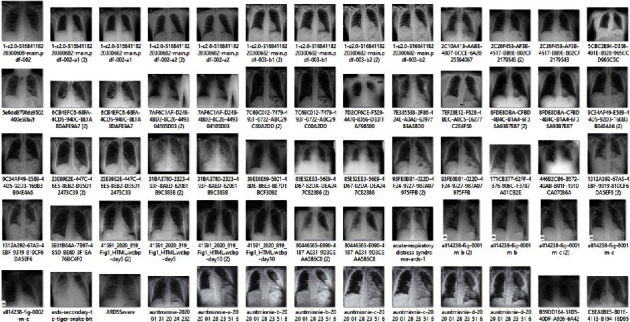
Sample input dataset of the projected prototype for COVID-19 disease detection.

**Figure 5:**
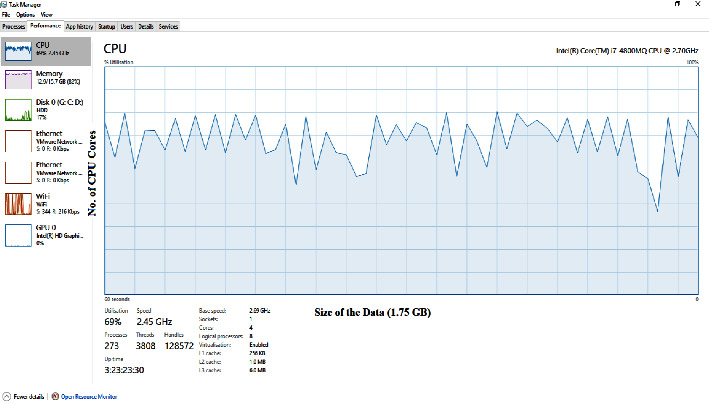
Processor and related resources occupancy of the computing device.

**Figure 6:**
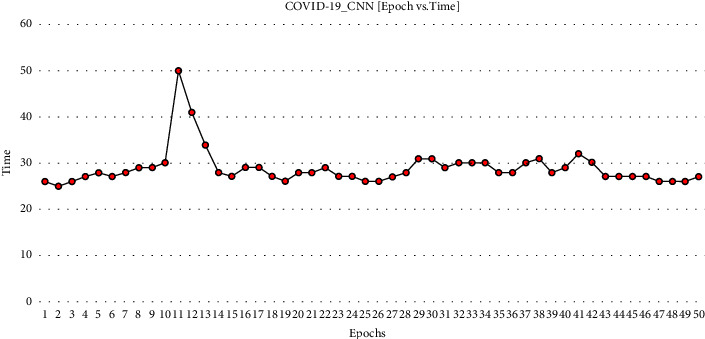
ECNN_COVID-19 graph comparing epochs vs. time.

**Figure 7:**
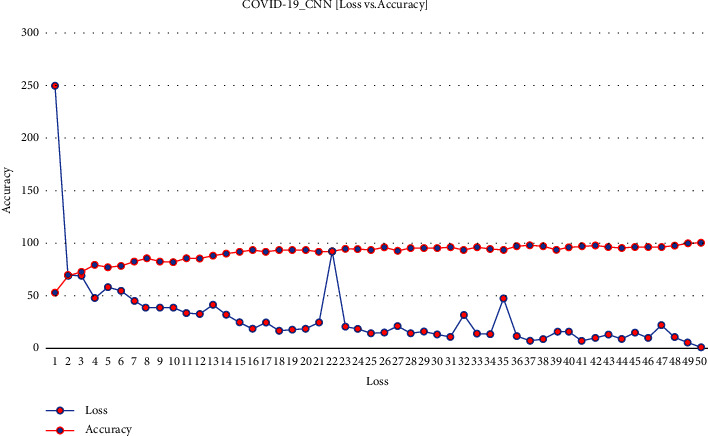
COVID-19 ECNN graph comparing accuracy vs. loss.

**Figure 8:**
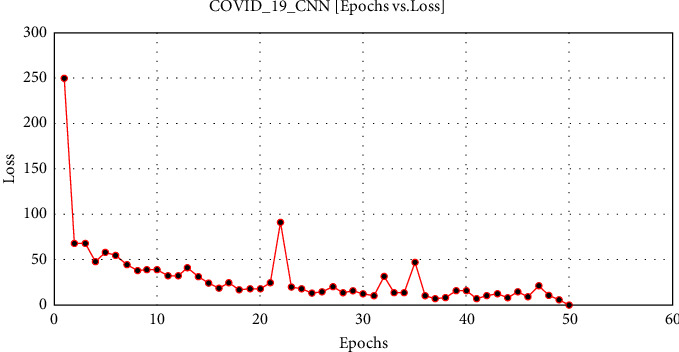
COVID-19 ECNN graph comparing epochs vs. loss.

**Figure 9:**
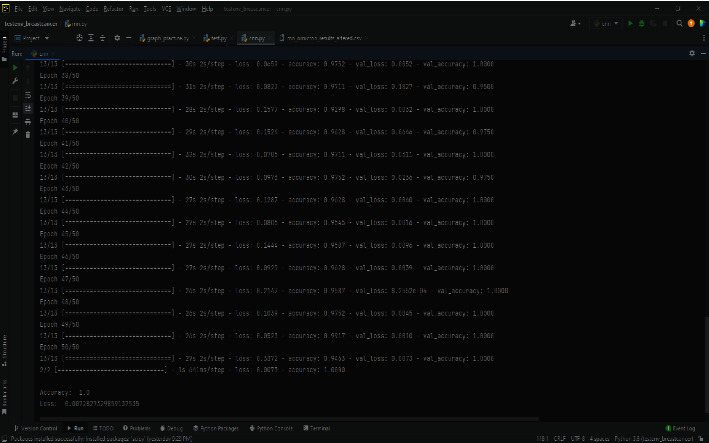
Final results of COVID-19 by using the ECNN approach.

The proposed prototype outperformed with an accuracy of 99.34% via using 39,256 X-ray image datasets Figure 3. The following section describes the findings of the research, which includes the algorithm steps Table 2, input dataset Figure 4, advantages of the proposed prototype compared against existing techniques Table 1, and final results obtained by using the proposed prototype against various metrics such as time consumption Figures 5 and 6, accuracy Figure 7, loss Figure 8, precision, etc. through demonstrated figures from Figures 1–3 and 5–9.

### 4.1. ECNN Algorithm

**Figure 10:**
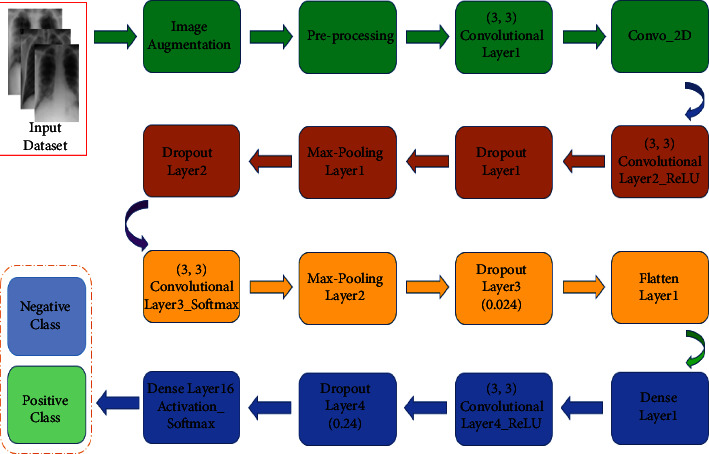
Block diagram of proposed ECNN technique.

**Figure 11:**
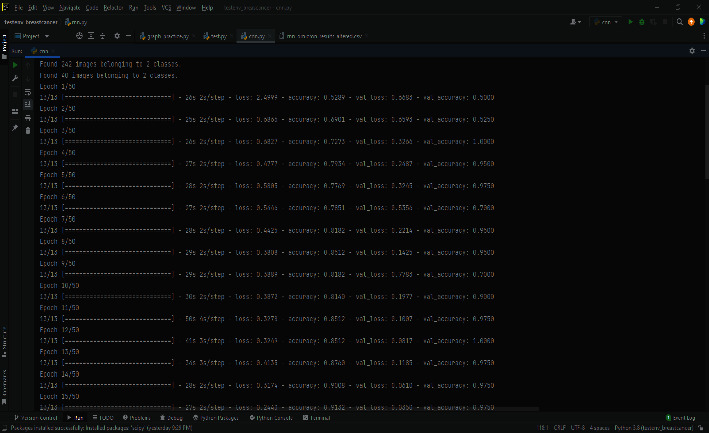
ECNN code performance stream.

Two trials of one or the other CC or MLO seen should be adjusted utilizing the picture enlistment method. At that argument, a dissimilar picture was received by removing the former trial from the existing trial and subsequently scaling to the full-range force. The territorial pictures from the refined district proposition are trimmed from the three pictures and scaled to 224 × 224 × 3 for each picture, which are utilized for ECNN floodlight extraction. As stated in Figure 10, the three channels are rehashed from one-channel grayscale pictures (e.g., the current sweep of 224 × 224 × 1) since the pertained ECNN and ECNN models expect 3-channel pictures. Multimeasurements of three-state in floodlights (from the earlier sweep, current output, and contrast pictures) are made to prepare a CNN model. For instance, The ECNN is floodlights utilizing ResNet-60V3 of 2048 × 3 measurements for each view (CC or MLO) of a subject's side (left or right bosom). Remember that the earlier sweep consistently relates to the ordinary (sound) status in any event for a destructive subject. According to Table 2, assuming we code sound and carcinogenic as 0 and 1 individually, at that point, the ground realities (yields) compared with the three states (earlier, current, distinction) of the destructive view are [0 1 1] Figure 11. This coding instrument can be handily stretched out to at least two earlier sweeps.

### 4.2. Algorithm

The following are the ECNN algorithm steps:

The COVID-19 disease infection data index, i.e., the absolute 39,256 pictures, of our experiment involved the related following steps:

1. Introduces mandatory collection
2. Introduces training dataset
3. Executes in the floodlight ordering of change data
4. Alignment with 70-time segments and 2 yield
5. Introduces Keras (Keras is a deep learning library)
6. Resets ECNN
7. Enhances ECNN part and regulates loss calculation function.
8. Improvement of yield part.
9. Adds the ECNN
10. Adjusts ECNN in the assessment dataset
11. Loads the COVID-19 disease infection test image data for the year 2020
12. Predict COVID-19 disease severity
13. Envision delayed consequences with expected or real Coronavirus illness contamination

Input Dataset: the input dataset has 16 columns with the target class as severity level of the COVID-19 disease and Figure 4 consists of 39,256 X-ray images with an average of 2111 pixels X 1509 pixels (width X height).

The ECNN calculation benefits: the benefits are as follows:

- Tremendous accuracy
- A lesser measure of time
- Less implementation time
- Less fault frequency
- Enormous data amount

The proposed block diagram Figure 10 demonstrates the number of input layers, hidden layers, dropout layers, pooling layers, dense layers, activation layers, and convolutional layers to predict and forecast the targeted output.

## 5. Discussion of Results

The outcomes of COVID-19 disease detection and prediction by incorporating X-ray images dataset with the ECNN technique are illustrated through figures

Figure 11 shows the execution flow of the ECNN code on COVID-19 database analyzing time taken, accuracy, loss, Val_Loss, and Val_Accuracy with respect to epochs.

The proposed model in Figure 9 achieves an accuracy of 99.34% on the database collected and used from Kaggle and UCI repositories.

The image in Figure 5 displays the CPU and related resources occupancy of the computer during ECNN code execution on the COVID-19 database.

### 5.1. Performance Evaluation Methods

The general trial result is estimated and introduced utilizing the most broadly utilized factual methodologies, for example, exactness, accuracy, review, F1-score, responsiveness, and particularity. For study sne, because of the restricted examples, the generally speaking measurable outcomes are addressed with a 95% certainty stretch followed by recently revealed writing that likewise utilized a little dataset [13, 23]. In our dataset, Omicron may be delegated genuine positive (Tp) or genuine negative (Tn), assuming people are analyzed precisely, and it very well may be characterized as bogus positive (Fp) or misleading pessimistic (Fn) if misdiagnosed. The assigned measurable measurements are made sense of in subtleties beneath.

Accuracy: the exactness is the general number of effectively recognized occasions across all cases. Utilizing the accompanying recipes, precision is not entirely settled.

(1) $$\begin{array}{c} \text{Accuracy}=\frac{\text{Tp}+\text{Tn}}{\text{Tp}+\text{Tn}+\text{Fp}+\text{Fn}}\text{.} \end{array}$$

Precision: precision is evaluated as the proportion of precisely anticipated positive results out of completely anticipated positive results.

(2) $$\begin{array}{c} \text{Precision}=\frac{\text{Tp}}{\text{Tp}+\text{Fp}}\text{.} \end{array}$$

Recall: recall alludes to the proportion of significant results that the calculation precisely distinguishes.

(3) $$\begin{array}{c} \text{Recall}=\frac{\text{Tp}}{\text{Tn}+\text{Fp}}\text{.} \end{array}$$

Sensitivity: sensitivity alludes to the main exact positive metric comparative with the complete number of events and can be estimated as follows:

(4) $$\begin{array}{c} \text{Sensitivity}=\frac{\text{Tp}}{\text{Tp}+\text{Fn}}\text{.} \end{array}$$

Specificity: it distinguishes the quantity of precisely recognized and determined genuine negatives and can be tracked down utilizing the accompanying recipe:

(5) $$\begin{array}{c} \text{Specificity}=\frac{\text{Tn}}{\text{Tn}+\text{Fp}}\text{.} \end{array}$$

F1-score: the F1 score is the symphonious mean of accuracy and review. The greatest conceivable F score is 1, which shows an amazing review and accuracy (99.34%).

(6) $$\begin{array}{c} F1-\text{Score}=2\times \frac{\text{Precision}\times \text{Recall}}{\text{Precision}+\text{Recall}}\text{.} \end{array}$$

Area under curve (AUC): the region under the bend (AUC) addresses the way of behaving of the models under different circumstances. AUC can be determined using the following equation:

(7) $$\begin{array}{c} \text{AUC}=\frac{\Sigma ri\left(Xp\right)-Xp\left(\left(Xp+1\right)/2\right)}{Xp+Xn}\text{.} \end{array}$$

### 5.2. Evaluation Methods

The following are measurements of evaluation methods or metrics.

(8) $$\begin{array}{c} \text{Quality}=\frac{\text{BP}+\text{VM}}{\text{BP}+\text{VP}+\text{BM}+\text{VM}}\text{,}\\ \text{Preciseness}=\frac{\text{BP}}{\text{BP}+\text{VP}}\text{,}\\ \text{Callback}=\frac{\text{BP}}{\text{BP}+\text{VM}}\text{,}\\ F-\text{measure}=\frac{2\times \text{Preciseness}\times \text{Callback}}{\text{Preciseness}+\text{Callback}}\text{.} \end{array}$$

### 5.3. Input Dataset

The input dataset has 16 columns with target class, i.e., the severity level of the COVID-19 disease, and consists of 39,256 X-ray images. The X-ray image(s) resolution of the input dataset is as follows: Width 2111 pixels x Height 1509 pixels.

The graph Figure 6 demonstrates the time taken to complete the iteration of epochs.

Figure 8 explicates the loss ratio accordant with each epoch during execution.

Figure 3 demonstrates the accuracy (99.34%) achieved against each epoch during execution.

Figure 7 demonstrates how the loss is reduced and accuracy gained from the training model of ECNN with respect to each iteration.

Value loss and value accuracy gained from the ECNN model during training are explained in Figure 1.

At a glance representation of the comparison among epochs, loss, accuracy of the ECNN model is described in Figure 2.

### 5.4. Comparison Table

Table 1 explains the comparison of the proposed technique with respect to various parameters in the existing system.

Table 1 explains the comparison factors of both techniques based on multiple parameters.

This research proposes research gaps in the existing system, which are less efficient, high time-complexity prone, high execution-time system, produces high error rate, and has no support for big datasets. To bridge these gaps, the proposed model outperforms in terms of all and is highly efficient, has less time complexity, less execution-time system, produces fewer errors, and supports big datasets.

## 6. Conclusion

This method suggests how the present prototype may be beneficial for more than one task, specifically if it is far taken into consideration that the modified U-Net prototypes do now no longer have higher overall performance. Also, it is proven how X-ray images' noise may produce predisposition with inside the prototypes. Most metrics display photographs without dissection as higher for categorizing COVID infections. The additional evaluation suggests that even though benchmarks are higher, those prototypes are primarily created on totally seen diagnosis throughout lung's X-ray as clean proof of COVID-19, so actual correct prototypes ought to be centered on lung elements for classification. In this situation, dissection is desired for dependable outcomes by lowering this bias. The transfer is getting to know that changed into crucial of the outcomes offered. As proven categorized models, the use of this approach wants 40 and 50 epochs to converge, even as segmentation prototypes without modification were approximately 282. The sequence of prototypes was obtainable to decide COVID-19 disease in chest X-ray images with an overall precision of 99% by categorizing COVID-19-positive and COVID-19-negative images. In the meantime, solitary for the COVID-19 label, the method achieved an avergage of 98.58% accuracy through a metric based-deep analysis at the database for a threshold of 0.4. Changing the edge suggest a growth for the accuracy of proposed architecture by as much as 99%.

The segmentation work suggests an excessive opportunity of imparting more statistics to elements in all experimentations, concluding the unconventional outcomes through dissecting lungs and including statistics mixed with surrounding noise. The noise is related to wires used in medical equipment's, patient's gender, and/or age, making photographs without lungs have extra information for classification in those situations. Either destiny efficacy or the use of prototypes without lungs should have been the very best possibilities for mislabeling photographs due to errors. Further research studies are required of section diagnosis recognized by the expert radiotherapist to make sure that any noise is a causing object for biased results. It is likewise critical to spot that the outcomes offered do now no longer always suggest the identical overall performance in each database. For example, the used database was collected of Asian victims; different international sufferers might also additionally display minor facts seize modifications or diagnosis, assuming a higher type is wanted the use of international databases. In addition, setting apart the databases through gender will offer extra statistics at the prototype`s scope because the tiny tissues of the chest might also additionally cover elements of the lungs, and it is far unidentified in case or not that it is taken into consideration a partiality with inside the forecast of the prototypical. The future work of this proposed approach can further be extended and implemented in real-time healthcare facilities to detect and predict COVID-19 infection in less time.

## Figures and Tables

**Table 1:** Comparison metrics with the existing system and ECNN technique.

Sl. No.	Name of the parameter	CNN	ECNN
1.	Accuracy	88.28%	99.34%
2.	Error rate	0.46	0.13
3.	Val_Loss	4.62	3.94
4.	Val_Accuracy	0.79	0.67
5.	Size of the dataset	1.75 GB	1.75 GB
6.	No. of epochs	50	50
7.	Time-complexity	O(n^2^)	O(n^2^)
8.	Execution time	1370 ms	1129 ms

**Table 2:** ECNN proposed model steps.

Step 1: Import required libraries
Step 2: Preprocessing of the dataset
Step 3: Combined CNN with extended neurons
Step 4: Perform 10-folded cross-validation with 2 classes
Step 5: Import Keras deep learning library with all supported libraries
Step 6: Reset all parameters of ECNN
Step 7: Enhance the ECNN part and regulation of the loss calculation function
Step 8: Enhancement of yield part of 10-folded with 2 classes
Step 9: Accumulate the ECNN parameters
Step 10: Adjusting the ECNN in the preparation of model
Step 11: Load the COVID-19 disease infection image dataset
Step 12: Predicting the infection severity by classifying the dataset into 2 classes
Step 13: Outcome of the trained model and stop the model

## Data Availability

The data used to support the findings of this study are available from the corresponding author upon request (head.sp@bluecrest.edu.lr).

## References

[1] Arias-Garzón D., Alzate-Grisales J. A., Orozco-Arias S. (2021). COVID-19 detection in X-ray images using convolutional neural networks. *Machine Learning with Applications*.

[2] Reshi A. A., Rustam F., Mehmood A. (2021). An efficient CNNModel for COVID-19 disease detection based on X-ray image classification. *Complexity*.

[3] Sarki R., Ahmed K., Wang H., Zhang Y., Wang K. (2022). Automated detection of COVID-19 through convolutional neural network using chest x-ray images. *PLoS One*.

[4] Aggarwal C. C. (2020). *Neural Networks and Deep Learning*.

[5] Ai T., Yang Z., Hou H. (2020). Correlation of chest CT and RT-PCR testing for coronavirus disease 2019 (COVID-19) in China: a report of 1014 cases. *Radiology*.

[6] Cennimo D. J. (2020). Coronavirus disease 2019 (COVID-19) clinical presentation. *Medspace*.

[7] Apostolopoulos I. D., Mpesiana T. A. (2020). Covid-19: automatic detection from X-ray images utilizing transfer learning with convolutional neural networks. *Physical and Engineering Sciences in Medicine*.

[8] Medical Imaging Databank of the Valencia region BIMCV (2020). BIMCV-Covid19-BIMCV. https://bimcv.cipf.es/bimcv-projects/bimcv-covid19/{#}1590859488150-148be708-c3f3.

[9] Bravo Ortíz M. A., Arteaga Arteaga H. B., Tabares Soto R. (2021). Clasificación de cáncer cervical usando redes neuronales convolucionales, transferencia de aprendizaje y aumento de datos. *Revista EIA*.

[10] Cucinotta D., Vanelli M. (2020). WHO declares COVID-19 a pandemic. *Acta BioMedica: Atenei Parmensis*.

[11] Rustam F., Reshi A. A., Mehmood A. (2020). COVID-19 future forecasting using supervised machine learning models. *IEEE Access*.

[12] (2020). X-ray (Radiography)-Chest. https://www.radiologyinfo.org/en/info.

[13] Li Q., Cai W., Wang X., Zhou Y., Feng D. D., Chen M. Medical image classification with convolutional neural network.

[14] Guan W. j, Ni Z. Y., Hu Y. (2020). Clinical characteristics of coronavirus disease 2019 in China. *New England Journal of Medicine*.

[15] Ghaderzadeh M., Eshraghi M. A., Asadi F. (2022). Efficient framework for detection of COVID-19 Omicron and delta variants based on two intelligent phases of CNN models. *Computational and Mathematical Methods in Medicine*.

[16] Srinivasulu A. (2021). Early prediction of covid-19 using modified recurrent neural networks. *Journal of Infectious Diseases and Treatment*.

[17] Cohen J. P. (2020). Github Covid19 X-ray dataset. https://github.com/ieee8023/covid-chestxray-dataset.

[18] Wang W., Xu Y., Gao R. (2020). Detection of SARS-CoV-2 in different types of clinical specimens. *JAMA*.

[19] Chen Z.-H. (2020). Mask-RCNN detection of COVID-19 pneumonia symptoms by employing Stacked Autoencoders in deep unsupervised learning on Low-Dose High Resolution CT. *IEEE Dataport*.

[20] Irfan M., Iftikhar M. A., Yasin S. (2021). Role of hybrid deep neural networks (HDNNs), computed Tomography, and chest X-rays for the detection of COVID-19. *International Journal of Environmental Research and Public Health*.

[21] Sitaula C., Aryal S. (2021). New bag of deep visual words based features to classify chest x-ray images for COVID-19 diagnosis. *Health Information Science and Systems*.

[22] Liu Y., Whitfield C., Zhang T., Hauser A., Reynolds T., Anwar M. (2021). Monitoring COVID-19 pandemic through the lens of social media using natural language processing and machine learning. *Health Information Science and Systems*.

[23] Almalki Y. E., Qayyum A., Irfan M. (2021). A novel method for COVID-19 diagnosis using artificial intelligence in chest X-ray images. *Healthcare*.

